# Care

**Published:** 1890-08-23

**Authors:** 


### Words of Consolation.

### CARE.

There are two sorts of care which affect people's minds—the care which makes them anxious and troubled, and the care which leads them to regard and protect persons or things. Both of these kinds are expressed by St. Peter, when he says "casting all our care upon God, for He careth for you." Now the first kind of care—anxiety, and distress of mind is very hard to bear; many say worse than pain and sickness, and, indeed, when they continue for long both mind and body suffer also. We know that man is born to trouble as the sparks fly upwards; how, then, are we to stand against these constant trials which beset us? By casting all our care upon God, for He careth for us. He tells us in many parts of Holy Scripture how we may be happy, though things are against us. For instance, we cannot, perhaps, get on in the world; everything we put our hand to fails or turns out badly, while our neighbour with the same wages and opportunities is prosperous. It is bitter as gall and wormwood to us; we cannot help envying him. The spirit of God gives us the remedy in the 37th Psalm. We find there "Fret not thyself because of him who prospereth in his way"; "Rest in the Lord and wait patiently for Him," "Commit thy way unto Him." It may to our impatience seem a long time before He answers, but He will surely bring it to pass.

Again, we may have troubles which none about us know of, which, indeed, we keep jealously to ourselves. We have been deceived by one very dear to us who has betrayed our confidence, or failed us in our greatest need. Our self love is so mixed up with it and wounded that we could not bear a stranger to intermeddle with our grief. It is all the more worrying and sad because we cannot open our griefs to a fellow creature. This burden, too, will be sustained by the Lord if we cast it upon Him. He is the friend that is born for adversity, He is the friend that sticketh closer than a brother.

There are the cares of a family, the anxiety for the safety of those so dear to us; we shall never have any happiness, unless we can believe and act on the belief, that He gives His angels charge over us to keep us in all our ways. Then, again, some of us take up unnecessary cares, looking after people who would rather be left alone, and who resent the interference, because we think with the woman in the American story, that Providence cannot get on without us. This should be dropped as soon as possible, for there is all the difference between being helpful and kind in bearing other people's burdens, and in meddling where we have no business.

Amidst all the turmoils and troubles of the flesh, it is very soothing and refreshing to remember that God careth for us. He loves us dearly as the children of His hand; He watches over us, and spies out all our ways. He will find us food and raiment, if we are truly His children. Our Lord draws a lovely lessson for us from the birds of the air and the lilies of the field, which He bids us consider—that is, think about—for, though they neither toil nor spin, yet are fed by our Heavenly Father. Are ye not much better than they? We may then, and should be, careful for nothing. Not in the haphazard style of the spendthrift and the sluggard, who live from hand-to-mouth, as it is called, but which is generally at the expense of somebody else's hands or mouths, but in everything let your requests be made known unto God by prayer and supplication. If we are content with such things as we have, He hath said, I will never leave thee nor forsake thee.

